# A Novel zf-MYND Protein, CHB-3, Mediates Guanylyl Cyclase Localization to Sensory Cilia and Controls Body Size of *Caenorhabditis elegans*

**DOI:** 10.1371/journal.pgen.1001211

**Published:** 2010-11-24

**Authors:** Manabi Fujiwara, Takayuki Teramoto, Takeshi Ishihara, Yasumi Ohshima, Steven L. McIntire

**Affiliations:** 1 Department of Biology, Graduate School of Sciences, Kyushu University, Hakozaki, Higashi-ku, Fukuoka, Japan; 2 Department of Applied Life Science, Faculty of Biotechnology and Life Science, Sojo University, Ikeda, Kumamoto, Japan; 3 Ernest Gallo Clinic and Research Center, Department of Neurology, Programs in Neuroscience and Biomedical Science, University of California San Francisco, San Francisco, California, United States of America; University of California San Francisco, United States of America

## Abstract

Cilia are important sensory organelles, which are thought to be essential regulators of numerous signaling pathways. In *Caenorhabditis elegans*, defects in sensory cilium formation result in a small-body phenotype, suggesting the role of sensory cilia in body size determination. Previous analyses suggest that lack of normal cilia causes the small-body phenotype through the activation of a signaling pathway which consists of the EGL-4 cGMP-dependent protein kinase and the GCY-12 receptor-type guanylyl cyclase. By genetic suppressor screening of the small-body phenotype of a cilium defective mutant, we identified a *chb-3* gene. Genetic analyses placed *chb-3* in the same pathway as *egl-4* and *gcy-12* and upstream of *egl-4*. *chb-3* encodes a novel protein, with a zf-MYND motif and ankyrin repeats, that is highly conserved from worm to human. In *chb-3* mutants, GCY-12 guanylyl cyclase visualized by tagged GFP (GCY-12::GFP) fails to localize to sensory cilia properly and accumulates in cell bodies. Our analyses suggest that decreased GCY-12 levels in the cilia of *chb-3* mutants may cause the suppression of the small-body phenotype of a cilium defective mutant. By observing the transport of GCY-12::GFP particles along the dendrites to the cilia in sensory neurons, we found that the velocities and the frequencies of the particle movement are decreased in *chb-3* mutant animals. How membrane proteins are trafficked to cilia has been the focus of extensive studies in vertebrates and invertebrates, although only a few of the relevant proteins have been identified. Our study defines a new regulator, CHB-3, in the trafficking process and also shows the importance of ciliary targeting of the signaling molecule, GCY-12, in sensory-dependent body size regulation in *C. elegans*. Given that CHB-3 is highly conserved in mammal, a similar system may be used in the trafficking of signaling proteins to the cilia of other species.

## Author Summary

*Caenorhabditis elegans* is a 1–2 mm long nematode. Its body size is controlled by sensory inputs; some mutants with defects in sensory perception grow into small size (20%–30% decrease in body volume), although the animals seem to feed normally. The EGL-4 cGMP-dependent protein kinase and the GCY-12 guanylyl cyclase act in sensory neurons to control body size downstream of sensory inputs. GCY-12 is localized to cilia, antenna-like cellular structures of sensory neurons. In the cilia, a number of signaling molecules are localized. Dysfunction of cilia is known to cause several human disorders such as Bardet-Biedl syndrome, illustrating the importance of these organelles. In this study, we identified a novel protein, CHB-3, involved in sensory-dependent body size regulation. Our analyses suggest that CHB-3 protein regulates the trafficking of GCY-12 from the cell bodies to the cilia. Without CHB-3 protein, GCY-12 fails to localize to cilia and body size is not controlled properly. Thus, the cilia are a special place for sensory information processing in body size regulation. Our analyses identified CHB-3 as a novel trafficking regulator of ciliary protein(s).

## Introduction

Cilia are highly conserved microtubule-based hair-like organelles, which are specialized subcellular compartments where a number of transmembrane receptors and channels are localized [1]. Through these molecules in cilia, environmental signals are transmitted to the cytoplasm and nucleus. For example, the outer segments of photoreceptor cone and rod cells in vertebrates are developmentally derived from cilia and the first steps of photosensation occur within the cilium. In addition to such classic sensory cilia, most vertebrate cells have a non-motile primary cilium, which also has a sensory function [2]. Dysfunction of the primary cilia causes a plethora of developmental disorders (ciliopathies) [3], illustrating the importance that these organelles play in signal transduction.

C. *elegans* is an excellent model system with which to understand how cilia form and function. Many mutants with defects in generation of normal sensory cilia have been isolated and the analyses of these mutants and the responsible genes have revealed the mechanism of cilium generation [4]. Interestingly, most of these mutants which lack normal sensory cilia exhibit a small-body phenotype, suggesting that sensory cilia are involved in the regulation of body size in C. *elegans* [5]. We have previously shown that the small body size of cilium-defective mutants is not due to an inability to locate food and that body size is regulated by a subset of amphid sensory neurons [6]. In order to identify the molecular mechanisms underlying sensory regulation of body size, we conducted a genetic screen for suppressors of the small body size of a cilium-defective mutant, *che-2*. These suppressors are termed Chb mutants for suppressor of *che-2* small body size. One of the Chb mutants, *egl-4*, exhibits a large-body phenotype and the body size is not decreased when a *che-2* mutation is introduced, indicating that cilium defects cause the small body size through *egl-4* function [6]. The *egl-4* gene encodes a cGMP-dependent protein kinase (PKG) [6]–[8]. EGL-4 appears to regulate body size by functioning in sensory neurons, because EGL-4 expression in several sensory neurons is sufficient for growth to a normal body size [6].

By genetic analyses, the DBL-1/TGF-β signaling pathway, which regulates hypodermal ploidy and consequently affects cell growth and body size [9], was shown to act downstream of EGL-4 [6], [7]. Indeed, *che-2* and *egl-4* mutants exhibit low and high ploidy of the hypodermal cell, respectively [10]. Because EGL-4 PKG can regulate gene expression by antagonizing a histone deacetylase [11], it is possible that EGL-4 kinase, under the control of sensory inputs, regulates body size by transcriptional regulation of such humoral factors as DBL-1/TGF-β.

Hence, lack of normal sensory cilia affects a signal transduction pathway of EGL-4 kinase and ultimately changes body size of *C. elegans*. However, the mechanisms how the EGL-4 activity is controlled through the cilia remain to be established. In this study, we establish that CHB-3 is a new upstream component of EGL-4 kinase that regulates body size. CHB-3 is a zf-MYND motif protein conserved from *C. elegans* to humans. We show that CHB-3 mediates the cilium localization of a guanylyl cyclase, GCY-12, and consequently regulates EGL-4 cGMP-dependent protein kinase activity. In a *chb-3* mutant, the frequency and the velocity of the dendritic transport of GCY-12 to cilia seem to be decreased, compared to those of wild-type animals. Our findings suggest that appropriate control of body size requires that certain signaling components, such as GCY-12, are localized to cilia (or their basement regions) to activate their proper targets (such as EGL-4), highlighting the indispensable role of sensory cilia as signaling centers.

## Results

### *chb-3* suppresses the small-body phenotype in the *che-2* mutant

**Figure 1:**
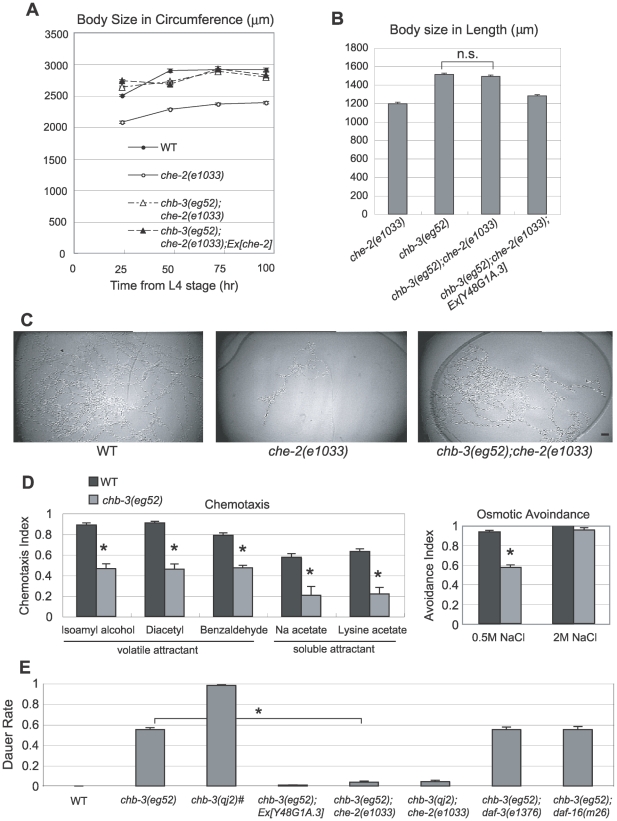
*chb-3* is a *che-2* small-body-size suppressor. (A) Time course of changes in body size indicated by the length of the perimeter of the lateral image of the animals. Body size was measured at the indicated time points from the L4 stage. Error bars indicate the standard error of the mean (s.e.m). (B) Body size indicated by the length of the animals 48 hours after the L4 stage. “n.s.” indicates that the difference is not significant (p>0.05, t test). (C) Typical tracks left by a single animal on an *E. coli* lawn during an 18-hours period. Scale bar, 10 mm. (D) Chemotaxis and osmotic avoidance assays. Each data point represents an average of at least 3 independent assays. Error bars indicate s.e.m. The mark (*) indicates the significant difference (p<0.01, t test). (E) The rate of dauer formation at 20°C. The Daf-c phenotype of *chb-3* was suppressed by *che-2(e1033)* but not by *daf-3(e1376)* or *daf-16(m26)*. Rescue of the *chb-3(eg52)* Daf-c phenotype by the Y48G1A.3 transgene (Ex[Y48G1A.3/*myo-3::gfp*]) is also shown. Each data point represents an average of at least 5 independent assays. Error bars indicate s.e.m. The mark (*) indicates the significant difference from *chb-3(eg52)* (p<0.01, t test). #The dauer formation rate of *chb-3(qj2)* was determined by gathering eggs from the *chb-3(qj2)*; Ex[*tax-4p*::CHB-3::GFP] rescued line, and observing hatched animals that lost the Ex array.

**Figure 2:**
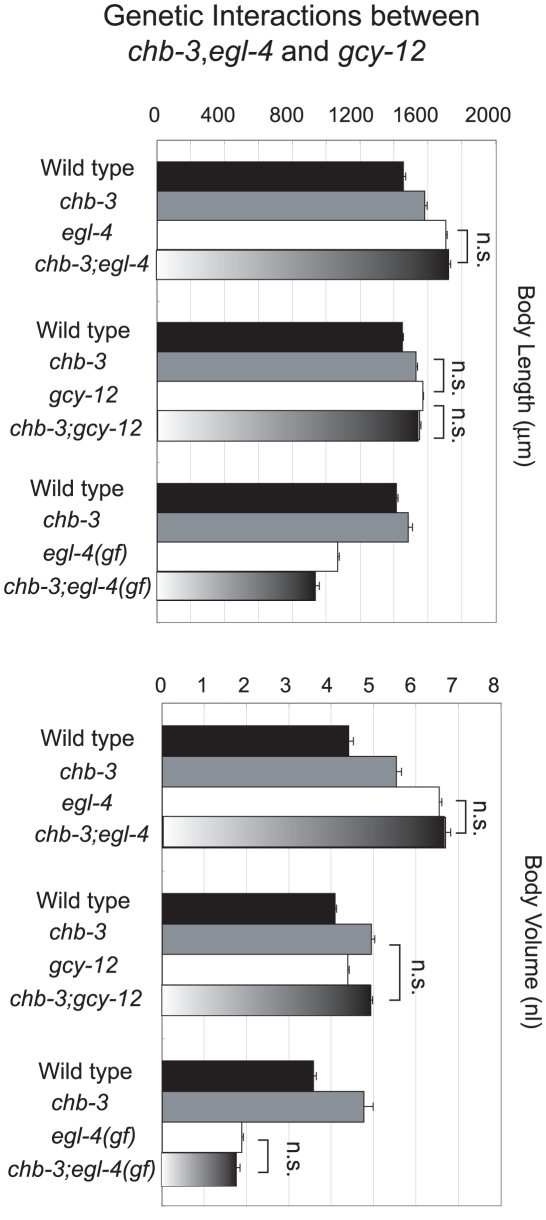
Genetic interactions among *chb-3*, *egl-4*, and *gcy-12*. Body size, indicated using the length (µm) and volume (nl), of animals 48 hours after the L4 stage. Error bars indicate s.e.m. “n.s.” indicates that the difference is not significant (p>0.05, t test).

*chb-3(eg52*) was previously isolated as a mutant which suppresses the small body size of *che-2*, but not the cilium structural defect of *che-2*, in a screen that also identified *egl-4* [6]. As shown in Figure 1A and 1B, the *chb-3(eg52)* mutation caused an increase in body size of *che-2(e1033)*. Compared with wild-type animals (WT, N2 strain), the *chb-3(eg52)* single mutant were longer and larger overall (Figure 2). Suppression of the *che-2* small-body phenotype by *chb-3*, however, is unlikely a result of simple additive effects of the mutation, because a *che-2* transgene did not increase the size of the *chb-3(eg52)*;*che-2(e1033)* mutant (*chb-3(eg52)*;*che-2(e1033)*;*Ex[che-2]* in Figure 1A). Moreover, the *chb-3(eg52)* single mutant and the *chb-3(eg52)*;*che-2(e1033)* double mutant were similar in body size (Figure 1B). We defined the group of Chb mutants displaying body sizes that were not affected by the *che-2* mutation as class I mutants, whereas the other Chb mutants were designated as class II mutants [6]. On the whole, the class I Chb genes are required for animals to make the body size small when animals do not perceive sensory inputs (e.g., cilium-defective mutants). The epistasis of *chb-3* to *che-2* indicates that *chb-3* is a class I Chb mutant, suggesting a role for the *chb-3* gene in the sensory processing-mediated regulation of body size.

We have previously shown that all class I Chb mutants isolated to date, such as *egl-4*, suppress the characteristic locomotory defect of *che-2* mutants [6]. Normally, wild-type animals alternate between two locomotory states: roaming and dwelling. Roaming is characterized by a relatively higher speed of movement with less turning, whereas dwelling is characterized by a relatively slower speed of movement with more turning. *che-2* mutants exhibit a more confined tracking pattern owing to increased time dwelling and decreased time roaming. Like *egl-4*, *chb-3* suppressed this locomotory defect of *che-2*. The *chb-3(eg52)*;*che-2(e1033)* double mutant showed a wider tracking pattern compared with *che-2* (Figure 1C). This result is consistent with a role for *chb-3* downstream of sensory inputs.

### *chb-3* animals exhibit defects in sensory-mediated behaviors

To analyze the role of *chb-3* in sensory perception, we examined other sensory behaviors of the *chb-3(eg52)* mutant. We found that *chb-3(eg52)* exhibits defects in chemotaxis behavior to a variety of attractants and in osmotic avoidance behavior (Figure 1D). For instance, compared with wild-type animals, the *chb-3(eg52)* mutant showed muted responses to either volatile (isoamyl alcohol, benzaldehyde, diacetyl) or soluble (Na, lysine) attractants. Aversive responses to high osmolarity solutions (0.5 M NaCl) were also less pronounced in *chb-3(eg52)* animals, although the response returned to a wild-type level if the osmolarity was high enough (2 M NaCl).

Another Chb mutant, *egl-4*, also shows defects in sensory behaviors [12]. These sensory defects suggest an important role for these molecules in sensory signal transduction and are consistent with the hypothesis that EGL-4 and CHB-3 are involved in sensory information processing underlying body size control of *C. elegans*. However, the phenotypes of *chb-3* and *egl-4* mutant animals are not completely identical; *chb-3(eg52)* showed reduced responses not only to isoamyl alcohol and diacetyl but also to benzaldehyde, whereas *egl-4* mutants responded normally to benzaldehyde [12].

### *chb-3* belongs to the Daf-c group of mutants with defects in cGMP signaling

We noted that *chb-3(eg52)* also exhibits a dauer-constituitive (Daf-c) phenotype. *C. elegans* develops into a diapause larvae called dauer if the worm experiences harsh environmental conditions such as a low food supply, a high population density or high temperature during early development. Daf-c mutants form dauers independent of the environmental conditions. About 50% of *chb-3(eg52)* animals formed dauers under conditions in which wild-type animals never formed dauers (Figure 1E).

Previous studies revealed that Daf-c mutants can be classified into three groups, thereby defining three branches of a pathway that act in parallel to regulate dauer formation [13]–[15]. Group I Daf-c mutants including *daf-11* have defects in a cGMP-signaling pathway. For example, *daf-11* encodes a receptor-type guanylyl cyclase [16]. The group I Daf-c mutants were strongly suppressed by cilium-defective mutations such as *che-2*, suggesting that certain sensory inputs are required for dauer formation by the group I mutants [14]. The group II Daf-c mutants have been shown to carry mutations in genes encoding components of the DAF-7/TGF-β signaling pathway [17]–[19]. Their Daf-c phenotype is suppressed by mutations in the *daf-3* gene, which encodes a downstream Smad transcription factor in the TGF-β signaling pathway [14], [20]. Another group of Daf-c mutants is caused by defects in insulin signaling. The Daf-c phenotype of this group is known to be suppressed by mutations in the *daf-16* gene, which encodes a FOXO transcription factor acting downstream of insulin [21].

To determine the Daf-c group in which *chb-3* belongs, double mutant analyses were performed. The Daf-c phenotype of *chb-3(eg52)* was strongly suppressed by *che-2(e1033)*, but not by *daf-3(e1376)* or *daf-16(m26)* (Figure 1E). In addition, one of the group I Daf-c mutants, *daf-11(m47)*, did not exhibit a higher dauer rate when the *chb-3(eg52)* mutation was introduced (data not shown). These analyses suggest that *chb-3* is a group I Daf-c gene, which may be involved in cGMP signaling.

### Genetic analysis of the relationship among *egl-4*, *chb-3*, and *gcy-12*

We previously showed that the cGMP-dependent protein kinase EGL-4 regulates body size by acting downstream of sensory inputs in *C. elegans* [6]. Recently, we identified a receptor type guanylyl cyclase gene, *gcy-12*, as another class I Chb gene (manuscript in preparation). *gcy-12* mutations, like those in *egl-4*, cause an increase in body length and volume compared with wild-type animals. Importantly, the *gcy-12*;*egl-4* double mutant does not become larger than the *egl-4* single mutant, indicating that EGL-4 and GCY-12 act in a linear pathway for body size regulation. Therefore, we hypothesized that GCY-12 produces cGMP, which activates the EGL-4 cGMP-dependent protein kinase and regulates body size in *C. elegans*. Because *chb-3* appears to play a role in a cGMP signaling pathway for dauer regulation, we asked if CHB-3 acts in the same pathway as EGL-4 and GCY-12. The *chb-3* mutation does not show synergy with either *egl-4* or *gcy-12* for body size; the *chb-3(eg52)*;*egl-4(ky185)* and *chb-3(eg52)*;*gcy-12(ks100)* double mutants were as large as the single mutants (Figure 2). Although we cannot exclude the possibility that the synergic effect was not observed by a physical constraint in the double mutants, these results suggest that CHB-3, EGL-4, and GCY-12 may function in the same regulatory pathway of body size control. To determine whether CHB-3 acts downstream or upstream of EGL-4, we used an *egl-4 (gf)* allele. *egl-4 (ad450gf)* is a gain-of-function mutant of *egl-4*, which seems to produce a constitutively activated EGL-4 kinase, and exhibits opposite phenotypes of *egl-4(lf)* including a small-body phenotype [22]. The *chb-3(eg52)* mutation did not increase body size in the *egl-4(ad450gf)* background. The *chb-3(eg52)*; *egl-4(ad450gf)* double mutant animals exhibited the same body volume as *egl-4(ad450gf)* with the slightly shortened body length, indicating that activated EGL-4 does not require functional CHB-3 when producing a small-body phenotype (Figure 2). Therefore, CHB-3 likely acts upstream of EGL-4, such as a regulator of kinase activity.

### *chb-3* encodes a novel protein with a zf-MYND motif

**Figure 3:**
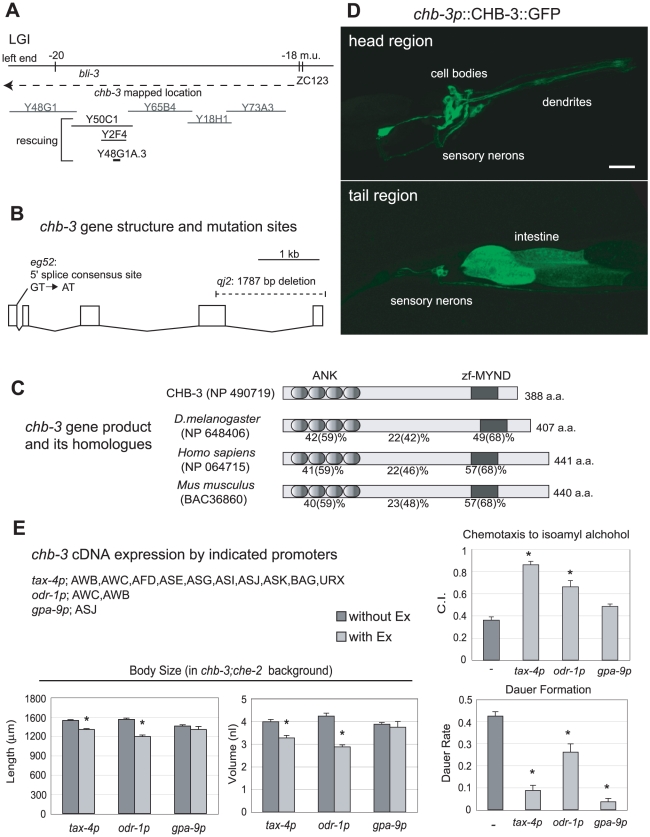
*chb-3* encodes a novel protein with a zf-MYND motif. (A) Genetic and physical map of the *chb-3* locus. Y50C1, Y2F4 (*C. elegans* genomic YAC clone), and a Y48G1A.3 PCR fragment (6.5 kb) which contained the Y48G1A.3 predicted gene and 1 kb of upstream sequence, rescued the Daf-c phenotype of *chb-3(eg52)*. The other clones are all YAC clones that failed to rescue the *chb-3* phenotypes. (B) Gene structure and mutation sites of *chb-3*/Y48G1A.3. Exons are boxed. (C) *chb-3* encodes a novel protein with four consecutive ankyrin repeats (ANK) close to the N terminus and a MYND-type Zn-finger motif (zf-MYND) close to the C terminus. Homologues from *D. melanogaster* (NP 648406), *Homo sapiens* (NP 064715), and *Mus musculus* (BAC36860) are included in the alignment. The amino-acid identities (and similarities in parentheses) to CHB-3 are shown for each ANK and zf-MYND domain and the region between the domains. (D) GFP expression from *chb-3p*::CHB-3::GFP in the head and tail regions of a wild-type animal (young adult stage). Projection of confocal microscopic sectioning images. Anterior is to the right. The scale bar represents 20 µm. (E) Phenotype rescue by *chb-3* expressed under the control of the indicated sensory promoters. Sensory neurons in which each promoter drives expression are also shown. To examine rescuing of the Chb phenotype (suppression of *che-2*-small-body size), each construct was introduced into *chb-3(eg52)*;*che-2(e1033)* animals as an extrachromosomal (Ex) array. Decrease of the size with the Ex array means rescuing of the Chb phenotype. To examine rescuing of the chemotaxis defect and Daf-c phenotype, each construct was introduced into *chb-3(eg52)* animals as an Ex array. Chemotaxis assays were performed with isoamyl alcohol (1/100 dilution). Animals with (light gray bars) and without (dark gray bars) the Ex array were compared. The mark (*) indicates the significant difference from control (p<0.01, t test).

By SNP mapping and germline transformation experiments, we identified a corresponding gene for the *chb-3(eg52)* mutation, Y48G1A.3 (Figure 3A, also see Materials and Methods). A genomic DNA fragment containing a single predicted gene (Y48G1A.3) restored the small body and the confined tracking pattern which are characteristic of the *che-2* single mutant, when introduced into the *chb-3(eg52)*;*che-2(e1033)* double mutant (Figure 1B and data not shown). The same fragment also rescued the Daf-c phenotype and the chemotaxis defect to diacetyl, when introduced into the *chb-3(eg52)* mutant (Figure 1E and data not shown). BLAST searches and conserved domain analysis (CDART) were performed with the predicted protein sequence of 388 amino acids. We found highly homologous proteins (∼30% identity/∼50% similarity throughout the protein) in species from flies to humans (Figure 3C), although none have defined functions. CDART revealed that CHB-3/Y48G1A.3 contains 4 consecutive ankyrin repeats in the N-terminal portion (amino acids 9-127). Ankyrin repeats are known to mediate protein-protein interactions in a diverse range of protein families. CHB-3/Y48G1A.3 also contains a motif termed a MYND type Zn-finger (zf-MYND) in the region close to the C terminus (amino acids 321-357). The zf-MYND motif is found in several different types of protein, including transcription factors and scaffolding proteins, and is thought to also mediate protein-protein interactions [23], [24].

### Mutational analysis of *chb-3(eg52)*

To identify the *chb-3(eg52)* mutation, we sequenced the entire coding region of Y48G1A.3, revealing a mutation in the 5′ splice consensus site of the 1st intron (GT to AT; Figure 3B). 5′ splice consensus is highly conserved in *C. elegans*, and this change may disrupt appropriate removal of the 1st intron from *chb-3* mRNA. By 5′ RACE analysis using total RNA prepared from *chb-3(eg52)* mutant animals, we confirmed that the 81-bp 1st intron, which is removed in mRNA isolated from wild-type animals, remained in *chb-3(eg52)* mRNA. This extra 81-bp sequence does not contain an in-frame stop codon, suggesting that the CHB-3 protein in *chb-3(eg52)* mutant animals contains 27 extra amino acids inserted into the middle of the 2nd ankyrin repeat. The *chb-3(eg52)* mutation is fully recessive and the mutant phenotypes can be rescued by introducing the wild-type *chb-3* gene, suggesting that *chb-3(eg52)* is a loss-of-function mutation. *chb-3(eg52)*, however, does not appear to be a null mutation. Therefore, we obtained a deletion mutation caused by Tc1 transposon excision in the *chb-3* gene. This deletion allele, *qj2*, lacks approximately 300 bp of coding sequence, including the region encoding the zf-MYND domain (Figure 3B). *chb-3(qj2*) showed a similar but more severe phenotype than *eg52*. As shown in Figure 1E, *qj2* exhibits a highly penetrant Daf-c phenotype, which can be suppressed by *che-2*. *qj2* also suppresses the *che-2* small-body phenotype as does *eg52* (data not shown). Because of the strong Daf-c phenotype, we could not analyze *qj2* for other phenotypes, such as chemotaxis behavior. The common phenotypes (Daf-c, Chb, and *gcy-12* localization pattern (see below)) and the molecular lesions in *eg52* and *qj2*, however, indicate that both *eg52* and *qj2* are loss-of-function mutants and that *qj2* is likely a null allele.

### *chb-3* function is critical in a subset of sensory neurons

We created a GFP reporter construct by using the *chb-3* promoter (2.3-kb upstream region) to express *chb-3* cDNA fused with gfp at the 3′ terminus (*chb-3p*::CHB-3::GFP). GFP expression was observed in many sensory neurons, including the amphid and phasmid neurons (Figure 3D). We also observed relatively weak expression in the intestine. GFP-fused CHB-3 appeared cytoplasmic and excluded from the nucleus (Figure 3D). To identify the tissues in which *chb-3* expression is required for normal function, *chb-3* cDNA was expressed in various sets of sensory neurons using several sensory neuron promoters (*tax-4p*, *odr-1p*, and *gpa-9p*) (Figure 3E) [25]–[28]. In these constructs, a GFP reporter gene was inserted into the 3′ end of *chb-3* cDNA for monitoring cellular expression and sub-cellular localization of CHB-3. Animals expressing these constructs were examined for dauer formation, chemotaxis, and suppression of the *che-2* small-body phenotype (Figure 3E). The *tax-4* promoter construct (*tax-4p*::CHB-3::GFP) rescued all the defects examined. In a *chb-3*;*che-2* background, *tax-4p*::CHB-3::GFP restored the *che-2*-like small-body phenotype (Figure 3E). In a *chb-3* background, *tax-4p*::CHB-3::GFP also restored chemotaxis to diacetyl and isoamyl alcohol, and decreased the rate of dauer formation (Figure 3E and data not shown). The *tax-4* promoter drives expression in 10 pairs of sensory neurons, suggesting an important role of *chb-3* in these sensory neurons (Figure 3E). CHB-3 expression was also limited to fewer sensory neurons using the *odr-1* and *gpa-9* promoters. In terms of the body size phenotypes, *odr-1p* expression showed rescuing activity (equivalent to *tax-4p*), suggesting that the AWB and AWC sensory neurons were involved in this regulatory process. For chemotaxis to isoamyl alcohol, none of these constructs rescued as strongly as was observed with *tax-4p*, and we were not able to identify a smaller subset of neurons in which CHB-3 specifically contributes to chemotaxis behavior. For the regulation of dauer formation, *chb-3* expression in the ASJ neuron is important, because the *gpa-9p* construct strongly rescued this phenotype. These results suggest that CHB-3 acts in a subset of sensory neurons to regulate the sensory processing required for multiple developmental and behavioral functions in *C. elegans*.

### The GCY-12 localization pattern is altered in *chb-3* mutants

**Figure 4:**
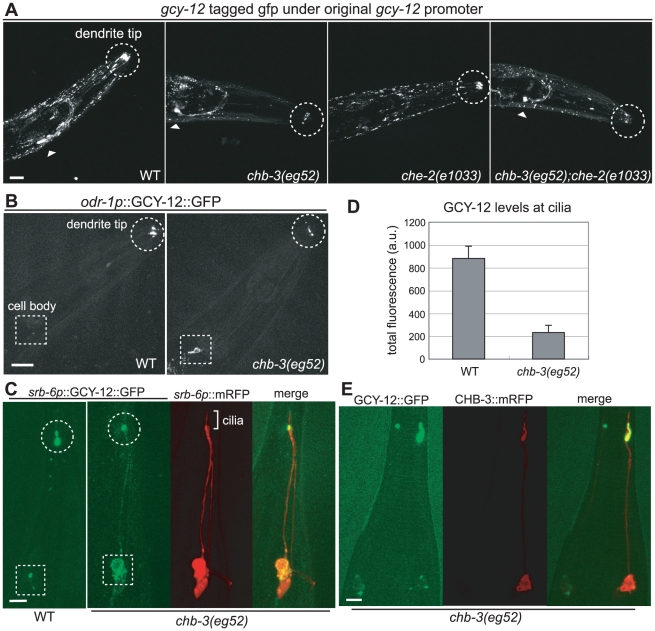
*chb-3* is required for proper localization of the GCY-12 guanylyl cyclase to cilia. (A) Expression of GFP-tagged, full-length GCY-12 under the control of the native promoter (GCY-12::GFP). Expression of the *ttx-3p*::mRFP injection marker in the AIY neurons is also shown (arrowheads). (B) Expression of GFP-tagged, full-length GCY-12 under the control of the *odr-1* promoter (*odr-1p*::GCY-12::GFP). (C) Expression of GFP-tagged, full-length GCY-12 under the control of the *srb-6* promoter (*srb-6p*::GCY-12::GFP). *srb-6p*::mRFP was also expressed to visualize the morphology of the cilia in *chb-3(eg52)*. (D) Ciliary GFP levels in animals expressing *odr-1p*::GCY-12::GFP were quantified using the confocal 3D analysis. Numbers of examined animals; wild-type (16), *chb-3* (18). Error bars indicate s.e.m. (E) Decreased GCY-12 levels in the cilia of *chb-3(eg52)* were rescued cell autonomously. The tail region of a *chb-3(eg52)* animal carrying two extrachromosomal transgenes, Ex[*srb-6p*::GCY-12::GFP] and Ex[*srb-6p*::CHB-3::mRFP], was shown. In this animal, Ex[*srb-6p*::CHB-3::mRFP] showed mosaic expression only in the right phasmid neuron, and only the right phasmid ciliary level of GCY-12::GFP was increased. (A, B, C, E) Projection of confocal microscopic sectioning images. Dendrite tips and cell bodies are marked with circles and squares, respectively. Head (A, B) and tail (C, E) regions of young adults are shown. Scale bars represent 20 µm (A, B) and 5 µm (C, E).

We observed GCY-12 expression and found that the GCY-12 localization pattern was changed in the *chb-3* mutant background. We fused a genomic fragment, which contains a 3-kb upstream region and the entire *gcy-12* coding region, with gfp at its 3′ end in frame (GCY-12::GFP). When the GCY-12::GFP construct was introduced into wild-type animals, GFP was strongly localized to the dendrite tip of sensory neurons, where cilia exist (Figure 4A). Punctuate GFP signals were also observed in head muscle tissue (Figure 4A). This expression profile likely reflected the actual expression pattern, because the construct retained the ability to rescue the *gcy-12* body size phenotype (data not shown). Next, we examined the *gcy-12* expression pattern in *chb-3* mutants. We transferred the extrachromosomal transgene, Ex[GCY-12::GFP], by mating from wild-type animals into a *chb-3* background, and found that the GCY-12 level in the dendrite tip was decreased in *chb-3* mutants (Figure 4A for *eg52*; Figure S1 for *qj2*). To observe the detailed localization pattern, we expressed *gcy-12* cDNA tagged with gfp in a limited number of sensory neurons; in AWC and AWB sensory neurons by using the *odr-1* promoter (*odr-1p*::GCY-12::GFP) and in PHA and PHB sensory neurons by using the *srb-6* promoter (*srb-6p*::GCY-12::GFP) [29]. AWC and AWB are the sensory neurons in which CHB-3 plays a vital role in body size regulation. PHA and PHB are neurons in the tail phasmid sensory organ with relatively short dendrites and morphologically simple cilia. In both sets of sensory neurons, GCY-12 at the dendrite tip was decreased in *chb-3(eg52)* (Figure 4B and 4C). In contrast, we found more GCY-12 accumulated in the cell bodies in many *chb-3(eg52)* animals (Figure 4B and 4C). We quantified the ciliary GFP levels in wild-type and *chb-3(eg52)* animals by 3D analysis with confocal microscopic images. The ciliary GFP level in *chb-3* animals expressing *odr-1p*::GCY-12::GFP was 26% of that of wild-type animals (Figure 4D). Based on the expression of *srb-6p*::mRFP, *chb-3(eg52)* retained normal PHA and PHB cilia, and GCY-12::GFP was localized to the bases of cilia which is called transition zone (Figure 4C).

Decreased GCY-12 levels in the cilia of *chb-3(eg52)* animals were rescued cell autonomously. By expressing CHB-3 protein tagged with mRFP by using the *srb-6* promoter (*srb-6p*::CHB-3::mRFP) and the *odr-1* promoter (*odr-1p*::CHB-3::mRFP), high GCY-12 levels at the cilia were restored in PHA/PHB and in AWC/AWB neurons, respectively (Figure 4E and Figure S2). Due to the mosaic expression of the extrachromosomal transgene, we observed an animal in which CHB-3 tagged with mRFP was expressed in a phasmid neuron in one side but not in a neuron in the bilaterally opposite side. In this case, high GCY-12 level was restored only in the cilia of the CHB-3 expressing neuron (Figure 4E, the neuron in the right side), but not in the cilia of the other neuron. These results indicate that CHB-3 functions to localize GCY-12 to the cilia cell autonomously.

Decreased GCY-12 levels at the cilia is not likely due to a decrease in *gcy-12* transcription, because the *gcy-12* 3-kb promoter fusion with gfp gave nearly the same expression level in wild-type and *chb-3(eg52)* animals (Figure S3). We also asked if ciliary membrane molecules other than GCY-12 require CHB-3 for their localization to cilia. We examined the expression of GFP-tagged constructs of a cyclic nucleotide-gated channel (CNG) TAX-2, and the G protein coupled receptors (GPCRs) ODR-10 (expressed under the control of its original promoter or an AWB-specific (*str-1p*) promoter) and SRD-1 (kindly gifted by Cori Bargmann). All of the examined proteins showed normal cilium localization in *chb-3(eg52)* animals, indicating that CHB-3 is not required for their cilium localization (Figure S4 and data not shown). The expression of GFP-tagged constructs also showed no apparent morphological defect of the examined cilia of *chb-3(eg52)* (Figure S4).

### CHB-3 regulates the dendritic transport of GCY-12::GFP

**Figure 5:**
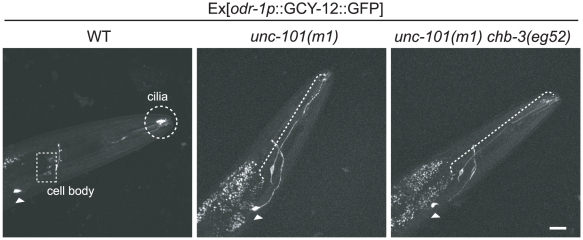
The UNC-101 clathrin adaptor AP-1 complex subunit is required for GCY-12 localization to cilia. Expression of *odr-1p*::GCY-12::GFP is indicated with broken lines. Expression of *ttx-3p*::mRFP (injection marker) in an AIY neuron is also shown (arrowhead). Projection of confocal microscopic sectioning images of head region in young adult. Scale bar represents 20 µm.

We attempted to analyze further the molecular function of CHB-3. *unc-101* is a gene encoding a protein related to the µ1 subunit of the AP-1 clathrin-coated vesicle adaptor complex, which is found in the trans-Golgi network (TGN) [30]. In *C. elegans*, *unc-101* is required for the localization of multiple ciliary membrane proteins, including several GPCRs and a receptor-type guanylyl cyclase, ODR-1 [31]. First, we examined the *odr-1p*::GCY-12::GFP expression pattern in a *unc-101* mutant. In *unc-101(m1)* null animals, GFP was uniformly distributed over the entire cell surface (Figure 5), indicating that GCY-12 as well as other ciliary membrane proteins is dependent on the somatodendritic sorting by UNC-101. Next, we examined the *odr-1p*::GCY-12::GFP expression pattern in the *chb-3(eg52) unc-101(m1)* double mutant, and found that the GCY-12 localization pattern was indistinguishable from that observed in the *unc-101* background; GFP was uniformly distributed over the entire cell surface in the double mutant (Figure 5). This suggests that CHB-3 acts after the somatodendritic sorting step in which UNC-101 is involved.

**Figure 6:**
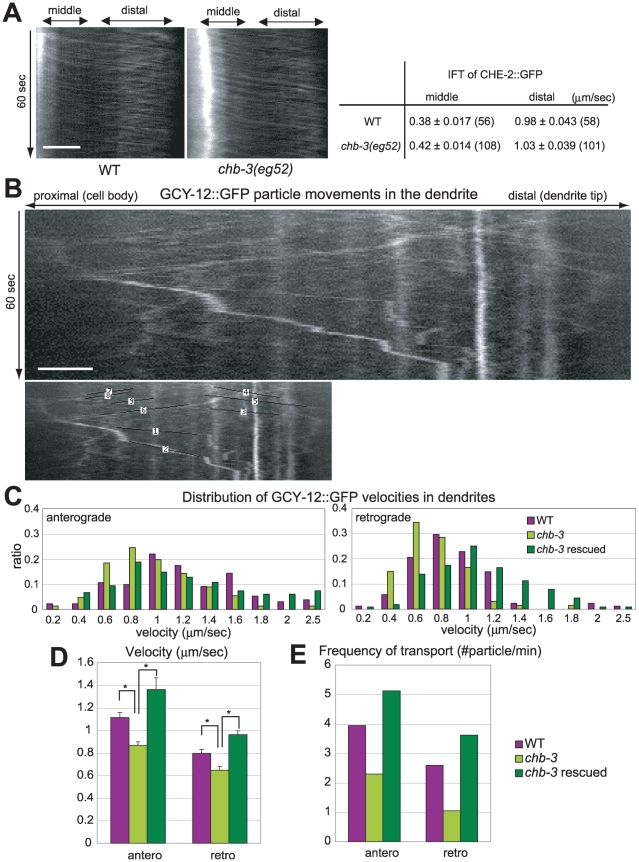
*chb-3* is required for proper GCY-12::GFP transport along the dendrites to cilia. (A) left; Kymographs depicting CHE-2::GFP particle motility within the cilia. right; The velocities of CHE-2::GFP particles along the middle segments and the distal segments of cilia are shown as the average ± s.e.m. (number of particles). (B) A kymograph depicting GCY-12::GFP particle motility along the dendrite of a phasmid sensory neuron in a wild-type animal. Corresponding lines, with which the velocity and the frequency were analyzed, are also shown. The movie is available upon requests. (A, B) The horizontal and vertical axes represent distance and time, respectively. Horizontal scale bar; 2 µm. (C) Distribution of GCY-12::GFP particle velocities in dendrites. (D) Average velocities of GCY-12::GFP particles in anterograde and retrograde directions in the dendrites with s.e.m. Asterisk indicates the significant difference at p<0.001 (t test). (E) Frequencies of GCY-12::GFP particles in anterograde and retrograde directions in the dendrites. (C, D, E) Wild-type, *chb-3(eg52)* and *chb-3(eg52)*;Ex[*srb-6p*::CHB-3::mRFP](*chb-3* rescued) animals were carrying Ex[*srb-6p*::GCY-12::GFP] transgene, and their dendrites of the phasmid neurons were observed. Total time of observation, total numbers of particles in anterograde direction and in retrograde direction are follows; WT (34 min, 134, 88), *chb-3* (64 min, 147, 67), *chb-3* rescued (32 min, 164, 116).

It is possible that CHB-3 may function in GCY-12 loading into the cilia and/or GCY-12 trafficking along cilia. Ciliary components, including several membrane proteins, are known to be transported from the base of the cilium to the tip of the cilium by the mechanism called the intraflagellar transport (IFT) [4], [32]. We examined if the *chb-3* mutation causes a defect in IFT and consequently affects the level of GCY-12 retained in the cilia. By using time-lapse fluorescence microscopy, we observed the movement of one of the IFT components CHE-2 tagged with GFP (CHE-2::GFP) in the cilia [33], [34]. CHE-2::GFP was properly localized to the cilia in *chb-3(eg52)* as was observed in wild-type animals. In the cilia of both wild-type and *chb-3(eg52)* animals, CHE-2::GFP moved relatively slowly (∼0.4 µm/sec) along the middle segments and was accelerated to ∼1 µm/sec along the distal segments in an anterograde direction (Figure 6A). Previously, it was reported that IFT along the middle and distal segments of cilia utilize distinct anterograde motor systems and exhibit different rates of IFT movements [35]. Our result is consistent with that and suggests that the *chb-3* mutant retains the normal IFT machinery. We did not detect the intraflagellar transport of GCY-12::GFP, most of which was accumulated at the bases of cilia, transition zone.

Next, we observed the dendritic transport of GCY-12::GFP. Because GCY-12::GFP appeared to be increased in the cell bodies of *chb-3* mutant animals, we examined if the *chb-3* mutation causes a defect in the dendritic transport of GCY-12 to the cilium base. First, we observed wild-type animals carrying Ex[*srb-6p*::GCY-12::GFP] transgene, and detected bidirectional movements of GCY-12::GFP particles along the dendrite of the phasmid sensory neurons by time-lapse fluorescence microscopy (Figure 6B). Depending on directionality, GCY-12::GFP particles move at different rates. Anterograde movement averages 1.11 (s.e.m. ±0.044, n = 134) µm/sec and retrograde movement averages 0.80 (s.e.m. ±0.036, n = 88) µm/sec (Figure 6C and 6D). These rates are comparable to those of the ODR-10 G-protein coupled receptor (anterograde: 1.42 µm/sec, retrograde: 0.71 µm/sec [31]) and those of the PKD-2 polycystin 2 channel (anterograde: 1.26 µm/sec, retrograde: 0.81 µm/sec [36]). Since the dendritic transport of these ciliary membrane proteins was observed in different sensory neurons (GCY-12 in PHA and PHB phasmid neurons, ODR-10 in AWB neurons, PKD-2 in CEM and RnB neurons), these results suggest that common dendritic transport machineries are used for membrane protein trafficking to cilia. Then, we analyzed the dendritic transport of GCY-12::GFP in the *chb-3(eg52)* mutant. We observed *chb-3(eg52)* carrying Ex[*srb-6p*::GCY-12::GFP] transgene, and found that GCY-12::GFP particle motility in the dendrites of the phasmid neurons was decreased (Figure 6C–6E and Figure S5). Compared to wild-type animals, the average velocities both in anterograde and in retrograde direction were lower in the *chb-3(eg52)* mutant (Figure 6D). Furthermore, the numbers of observed particles which move at least 3 µm in a steady rate were decreased both in anterograde and in retrograde direction in *chb-3(eg52)* (Figure 6E). *chb-3(eg52)* animals carrying not only Ex[*srb-6p*::GCY-12::GFP] but also Ex[*srb-6p*::CHB-3::mRFP] transgene (*chb-3* rescued) recovered motility and exhibited even higher velocities and frequencies of GCY-12::GFP particle movement than wild-type animals (Figure 6D, 6E, and Figure S5). These results suggest that CHB-3 regulates the dendritic transport of GCY-12::GFP, and that decreased levels of GCY-12::GFP in the cilia of *chb*-*3* may be caused by the defect in the dendritic transport.

### The decrease in ciliary GCY-12 levels may affect the body size

**Figure 7:**
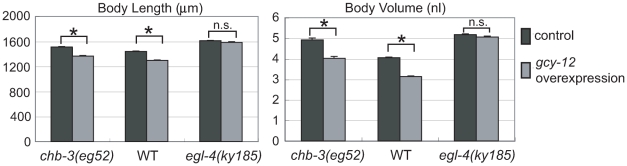
The effect of *gcy-12* overexpression on body size. Genomic DNA containing the entire coding region of *gcy-12* and its upstream region (3 kb) was introduced into the animals as an extrachromosomal array (Ex). Animals with (gray bars) and without (black) the Ex array were compared. Error bars indicate s.e.m. The mark (*) indicates the significant difference (p<0.005, t test), and “n.s.” indicates that the difference is not significant.

We examined whether decreased levels of GCY-12 in the cilia cause the *chb-3* body-size phenotypes. If the decrease in ciliary GCY-12 caused the *chb-3* large-body phenotype, overexpression of *gcy-12* may rescue the phenotype. Figure 7 shows the effect on body size of *gcy-12* overexpression by the original *gcy-12* promoter. Overexpressing the *gcy-12* gene indeed restored the *chb-3(eg52)* body size to a level comparable to wild-type animals. *gcy-12* overexpression also decreased the body size of wild-type animals, suggesting that *gcy-12* was acting in a dose-dependent manner. These GCY-12 overexpression effects were likely through modulation of EGL-4 activity, since overexpression did not affect the body size of the *egl-4(ky185)* mutant (Figure 7).

The suppression of the *che-2* small-body phenotype by mutations in *gcy-12* or *egl-4* indicates that the *che-2* small-body phenotype is caused by the functions of these molecules. The lack of normal cilia seems to cause inappropriate activation of this signaling pathway, which ultimately makes the body size small. As shown in Figure 4A, we found that GCY-12 was still localized to dendrite tips in the *che-2(e1033)* mutant. In the *che-2(e1033)* mutant, the distal and middle segments but not transition zone of the cilia are missing [5]. GCY-12::GFP may be localized to transition zone of the shortened cilia of the *che-2* mutant. On the other hand, GCY-12 in the dendrite tip was decreased in the *chb-3(eg52)*;*che-2(e1033)* double mutant, as was observed for the *chb-3* single mutant (Figure 4A). Because mutations in *chb-3*, as well as in *gcy-12* and *egl-4*, suppress the *che-2* small-body phenotype, these localization patterns suggest that GCY-12 needs to be at the cilia (or their bases) for activation of the GCY-12-EGL-4 signaling pathway. The shortened sensory cilia of *che-2* mutants may activate GCY-12 to induce the small-body phenotype, then *gcy-12* and *chb-3* mutations may suppress this phenotype by abolishing and decreasing GCY-12 protein levels in the cilia, respectively. Thus, the ciliary GCY-12 levels may affect body size, and CHB-3 is an important regulator of the localization processes.

## Discussion

Cilia are important sensory organelles, which are now thought to be essential regulators of numerous signaling pathways. In this paper, we identified *chb-3*, a new Chb (*che-2* small body size suppressor) gene, that is required for body size control through the cilia in *C. elegans*. Genetic analyses placed *chb-3* in the same pathway as *egl-4* (a gene encoding a cGMP-dependent protein kinase) and *gcy-12* (a gene encoding a receptor-type guanylyl cyclase), and probably upstream of *egl-4*. *chb-3* encodes a highly conserved protein from worms to humans, which contains ankyrin repeats and a zf-MYND motif. We found that CHB-3 acts cell autonomously and mediates GCY-12 localization to cilia through the regulation of the dendritic transport. We also showed that decreased ciliary levels of GCY-12 are a probable cause of the body size change observed in *chb-3*. These results reveal the role of CHB-3 in ciliary membrane protein trafficking, and highlight the important role of sensory cilia in body size determination.

### EGL-4 kinase is regulated positively and negatively at the cilia

Previously, we proposed a model in which sensory perception, acting through modulation of the GCY-12-EGL-4 pathway, regulates body size [6] (manuscript in preparation). Normally, activation of the GCY-12-EGL-4 pathway reduces body size. In wild-type animals, sensory inputs through cilia are likely to negatively regulate the GCY-12-EGL-4 pathway, since the loss of sensory inputs due to *che-2* mutations causes inappropriate activation of the GCY-12-EGL-4 pathway and results in a small-body phenotype. In this model, sensory stimuli inhibit, rather than stimulate sensory neurons, as is reported in several sensory signal transduction pathways [37], [38]. Here, we showed that CHB-3 functions in the localization of GCY-12 to cilia and plays an indispensable role in the activation of the GCY-12-EGL-4 pathway. Thus, GCY-12 apparently needs to be present at cilia (or transition zone of cilia) to activate the EGL-4 kinase, although EGL-4 is not restricted to cilia and is rather broadly distributed throughout the entire cytoplasmic region (as far as can be observed with tagged GFP constructs [6]). This is potentially because the signaling complex (which allows for effective signal transduction) may be formed at the cilia, or because GCY-12 may require ligand binding at the cilia to be activated. Hence, the cilium is likely to be a place where the EGL-4 activity is controlled positively (by GCY-12) and negatively (by sensory inputs through unknown molecules being dependent on normal cilia). The levels of EGL-4 kinase activity are a consequence of these opposing controls, which thereby determine the ultimate body size of *C. elegans*.

### CHB-3 acts in a small subset of sensory neurons to regulate of body size

*chb-3* is expressed in multiple neurons and the intestine of *C. elegans*. Rescue experiments with various sensory promoters suggested that CHB-3 functions in a small subset of sensory neurons; it is sufficient to express *chb-3* in two pairs of sensory neurons, AWC and AWB, for normal body size regulation. These neurons are included in a set of sensory neurons, EGL-4 expression in which is sufficient for normal body size regulation [6]. However, *egl-4* expression in AWC and AWB by the *odr-3* promoter is not sufficient to rescue the body size phenotype of the *egl-4* mutant [6]. This suggests that EGL-4 may function for body size regulation in other sensory neurons that do not require CHB-3, too. This is consistent with the observation that the *egl-4* mutant is larger than the *chb-3* mutant and the *gcy-12* mutant (Figure 2). EGL-4 may act dependently and also independently of CHB-3 in the regulation of body size.

### Other behavioral and developmental roles of CHB-3

*chb-3* mutants exhibit not only body-size phenotypes but also defects in chemotaxis and a group I type Daf-c phenotype, which are not shared by *egl-4* mutants [12] and *gcy-12* mutants (manuscript in preparation). It is possible that CHB-3 mediates the cilium localization of not only GCY-12 but also other signaling molecules that are required for chemotaxis and control of dauer formation. One such candidate is DAF-11, another receptor-type guanylyl cyclase that contributes to dauer regulation and chemotaxis and is localized to sensory cilia [16]. Both *chb-3* and *daf-11* are group I Daf-c mutants. Our analysis also indicates that the Daf-c phenotype of *chb-3* is strongly rescued if *chb-3* is expressed in ASJ sensory neurons, where *daf-11* acts in dauer control [39]. Strikingly, Jensen VL et al. are reporting that DAF-11 cilium localization is indeed controlled by a protein encoded by *daf-25* gene, which turned out to be an identical gene to *chb-3*/Y48G1A.3 (Jansen VL et al., 2010, in this issue). Thus, based on our and their results, CHB-3/DAF-25 mediates the cilium localization of at least two different receptor-type guanylyl cyclases of *C. elegans*. Although we observed that several other cilium membrane proteins, including GPCRs and CNG, localized to cilia independent of CHB-3, further analyses may reveal additional molecules that are dependent on.

#### CHB-3/DAF-25–mediated trafficking

Interestingly, we found that the *unc-101(m1)* mutation almost completely suppressed the Daf-c phenotype of the *chb-3(eg52)* mutant (Figure S6). We speculate that the *unc-101* mutation may bypass the requirement for CHB-3 by causing the re-localization of signaling components such as DAF-11 throughout the cell surface and consequently elevating the level of these molecules at cilia. If this were the case, *unc-101* would also suppress the body size phenotype of *chb-3* by distributing GCY-12 throughout the cell surface. We, however, failed to observe this, because the *unc-101(m1)* mutant itself has a small-body phenotype probably due to a muscle contraction defect.

### *chb-3* encodes a highly conserved zf-MYND protein that may act in the dendritic transport of ciliary membrane proteins

Recently, cilia have become the focus of intensive studies evaluating their contributions to the transduction of extracellular signals. It is clear that cilia play fundamental roles in several mammalian processes, including Sonic Hedgehog morphogen signaling and left-right axis determination [40]. Ciliary dysfunction also causes a number of human disorders (ciliopathies), including Bardet-Biedl syndrome (BBS) characterized by retinal degeneration, early-onset obesity and cognitive impairment [3]. Proteins mutated in BBS are shown to be involved in the regulation of membrane proteins trafficking into cilia with a small GTPase, Rab8 [41]. Rab8 and several other small GTPases regulate the trafficking of post-TGN vesicles to cilia [41], [42]. However, our understanding of how membrane proteins trafficking from Golgi to the cilium is regulated remains extremely limited [43]. Although only a small number of proteins involved in this process have been identified, the analyses of these proteins suggest that there is an active and highly directed mechanism for placement of membrane proteins to the cilia. IFT20 is a conserved protein which dynamically moves between the Golgi complex and the cilium, and is thought to act in the delivery of ciliary membrane proteins [44]. UNC-101 clathrin AP-1 µ1 subunit in *C. elegans* acts in the sorting or packaging of ciliary membrane proteins at trans-Golgi network [31]. In *C. elegans*, ODR-4 membrane protein is thought to act in odorant receptor folding or transport to cilia [45], and CIL-1 PI 5-phosphatase is required to confine PKD-2 polycystin 2 channel to the cilia [46].

Here, we show that CHB-3 may act in the dendritic transport of GCY-12 to the cilia. *chb-3* encodes a novel zf-MYND protein, which is highly conserved from *C. elegans* to humans. The zf-MYND motif, containing two putative zinc fingers, is found in several different types of proteins [47]. Some zf-MYND proteins act as histone deacetylase-dependent transcriptional repressors through binding a silencing mediator complex with their zf-MYND domains [23], [24], [48]–[50]. Another zf-MYND protein, *Drosophila* nervy, binds a cAMP-dependent protein kinase and the Plexin receptor, and controls neuronal axon guidance by integrating different guidance cues [51], [52]. Thus, the zf-MYND motif appears to mediate specific protein-protein interactions. CHB-3 also contains N-terminal ankyrin repeats, which are generally involved in protein-protein interactions. Two alleles of *chb-3*, *eg52* and *qj2*, are likely to produce a protein with disturbed ankyrin repeats and a protein lacking the zf-MYND domain, respectively. These alleles exhibit an equivalent defect in GCY-12 localization, indicating that both ankyrin repeats and the zf-MYND motif are important for this function. The homologous proteins of CHB-3 in other species, which also possess these two domains, might be also involved in dendritic transport.

In *unc-101* mutants, GCY-12 is evenly distributed over the plasma membranes. This may be because loss of AP-1 µ1 causes GCY-12 to enter a different class of vesicle at the TGN that can fuse anywhere in the plasma membrane. In the *chb-3 unc-101* mutant, the GCY-12 localization pattern was indistinguishable from that observed in the *unc-101* background, suggesting that *chb-3* mutations possibly disrupt steps following the TGN rather than processing occurring before the TGN. We observed that GCY-12::GFP particles move in the dendrites of sensory ciliated neurons. The velocities in anterograde and retrograde directions are comparable to the previously reported velocities of other ciliary membrane proteins in *C. elegans*. This indicates utilization of common trafficking machineries, although no motor protein such as dynein and kinesin has been identified for ciliary protein transport along dendrites in *C. elegans*. In the *chb-3* mutant, GCY-12::GFP particle motility in the dendrites is decreased; GCY-12::GFP particles move significantly slower and particle movements are observed less frequently. We did not detect dendritic movement of CHB-3::GFP, which was rather evenly distributed in the dendrite. Thus, CHB-3 itself does not appear to move along dendrites. Given that the CHB-3 protein possesses two protein-protein interaction domains, one possible function of CHB-3 is to associate the post-TGN vesicles containing GCY-12::GFP with dendritic transport machineries as a transient adaptor protein. Alternatively, it is possible that CHB-3 itself is a component of the dendritic transport machinery. In either case, CHB-3 seems to act not generally for all ciliary membrane protiens but specifically for a limited number of proteins including GCY-12. Future studies, including the search for proteins interacting with CHB-3 and their homologues in higher organisms, may fully reveal the molecular functions of this protein and mechanisms of ciliary targeting. The search for additional Chb mutations and analyses of their molecular functions may also lead to the identification of other regulators acting in the system.

Our study has identified a new regulator, CHB-3, of the trafficking of membrane proteins to cilia. We also demonstrate the importance of ciliary targeting of a guanylyl cycalse, GCY-12, in sensory-dependent body size regulation. Given that CHB-3 is highly conserved, a similar system might be used for the control of trafficking of signaling proteins in various species.

## Materials and Methods

### Strains and genetics

*C. elegans* strain N2 was used as wild-type animals. Worms were grown at 20°C with food, *E. coli* OP50, using standard methods [53].

The other strains used in this work included *chb-3(eg52)*, *chb-3(qj2)*, *gcy-12(ks100)*, *che-2(e1033)*, *egl-4(ky185)*, *daf-3(e1376)*, *daf-16(m26)*, *daf-11(m47)*, *bli-3(e767)*, and *unc-101(m1)*. Double mutants were generated using standard methods and confirmed by complementation testing or sequencing.

The *che-2*; *Ex[che-2(EcoT14I)/H20::gfp]* strain was kindly provided by Isao Katsura [33]. *Ex[che-2(EcoT14I)/H20::gfp]* (*Ex[che-2]*) is an extrachromosomal array containing a *che-2* gene with its original promoter, and retains a rescuing ability of the *che-2* small-body phenotype [6].

### Body size measurement

L4 crescent stage animals were transferred to 6-cm seeded NGM plates and maintained under well-fed conditions until the body size was measured. For body length and volume, animals were anesthetized with NaN_3_ 48 hours after the L4 stage and measured by using ‘Senchu-gazou-kaiseki-souchi SVK-3A’ (Showa Denki Co., Fukuoka, Japan) as described by Hirose et al. [7]. As another indicator of the body size, we measured the length of perimeter of the lateral image of the animals as previously described [6]. Each data point represents the average volume and length of more than 20 animals. All strains plotted on each graph were measured at the same time.

### Locomotory behavioral assay

All seeded plates for the locomotory behavioral assay were freshly spread with bacteria, incubated overnight at 37°C, and allowed to cool to room temperature before use. For the tracking assay; a single well-fed L4 crescent stage animal was transferred to an individual 3.5-cm seeded NGM plate, and allowed to move freely for 17–20 hours at room temperature. The assay plates were not moved during this period to avoid any mechanical stimulation.

### Dye-filling assay and other behavioral assays

Dye-filling assay; assays were done as described by Starich et al. using DiI (10 µg/ml) [54].

Osmotic avoidance; assays were performed basically as described by Culotti and Russell [55]. First, rings (1 cm in diameter) were drawn on plates with 50 µl of NaCl solution of indicated concentration. Then, 25 animals were placed inside of the ring. After 30 minutes, the animals inside and outside of the ring were counted. The avoidance index was calculated as the number of animals which stayed inside of the ring/the total number of animals.

Chemotaxis; assays were done as described by Bargmann [56]. One microliter of isoamyl alcohol (1/100 dilution), diacetyl (1/100 dilution) or benzaldehyde (1/100 dilution) for volatile attractants, and agar plugs (7 mm in diameter) soaked with sodium acetate (0.4 M) or lysine acetate (1 M) for soluble attractants, were used.

Dauer formation; eggs were gathered by bleaching well-fed adults, and placed on seeded plates (200–300 eggs/plate). After 50 hours at 20°C, the dauers and non-dauers were counted. The criteria for dauer were a dark intestine, long shape, suppressed pumping, and lethargic movements.

### Quantification of GCY-12–tagged GFP in cilia

Young adult animals (48 hours from the L4 stage) harboring Ex[*odr-1p*::GCY-12::GFP] were examined for fluorescence at the nose tip. An LSM510 confocal microscope (Carl Zeiss) equipped with a 100x objective lens was used to scan GFP fluorescence using a 488-nm excitation beam and a BP 500–550 nm emission filter. Nose tips were optically sliced in 0.7-µm intervals, and the stacked images were processed with “3D for LSM” software (Carl Zeiss) to calculate the total fluorescence at the nose tip (the product of the volume of the fluorescent area and the mean fluorescence). Each data point represents the average from 16-18 animals.

### IFT and dendritic transport analyses

Time-lapse imaging analyses were performed basically as described by Signor et al. [57]. Time-lapse images were obtained with Olympus IX71 microscope equipped with 100 x, 1.4 N.A. objective and ORCA-ER digital camera (Hamamatsu) with Uniblitz VMM-D1 shutter unit (Ancient Vassociates). All images were collected at 1 frame/0.5 sec intervals, and at 100–400 msec exposure time for 1 min duration, with adult hermaphrodite anesthetized with 10 mM levamisole. When animal's positions were not completely fixed by levamisole, those were digitally fixed by the custom registration software (written in Matlab). In the software, the algorithm minimizes the mean square difference between the pixel intensities of ROI (region of interest) of two consecutive time slices [58]. Then, movies and kymographs were created by using MetaMorph software. Animals were cultured at 20°C and observed at 22–23°C. The intraflagellar transport of CHE-2::GFP was analyzed with phasmid neurons of animals expressing CHE-2 tagged GFP driven under the *che-2* promoter [33]. Our IFT analysis of CHE-2::GFP in wild-type animals yielded velocities which are slightly slower than those in a previous report (anterograde middle segment; 0.7 µm/sec, anterograde distal segment; 1.3 µm/sec [59]). This may be due to the differences in the observing conditions. For the analysis of GCY-12::GFP dendritic transport, the same transgene (Ex[*srb-6p*::GCY-12::GFP]) was transferred to N2 and *chb-3(eg52)* by mating for comparison under the same expression level of GCY-12::GFP. As the “rescued *chb-3(eg52)*” line, Ex[*srb-6p*::CHB-3::mRFP] transgene was introduced into *chb-3(eg52)*; Ex[*srb-6p*::GCY-12::GFP], and animals carrying two transgenes were used. The frequencies of GCY-12 transport along dendrites were analyzed based on kymographs, and a path containing at least one straight segment corresponding to a movement longer than 3 µm in a steady rate was counted as 1 event. If a GCY-12::GFP particle changes the speeds or the directions in a path, the longest straight segment was analyzed for the representative speed and direction of the particle.

### Molecular biology methods

For the *chb-3* cDNA expression analysis, most of the *chb-3* cDNA was obtained from the yk280C9 cDNA clone (provided by Yuji Kohara). The 5′ region of the first exon was obtained using 5′ RACE (5′-RACE system, Invitrogen), and fused to the yk280C9 cDNA to create a full-length cDNA. A BamHI site just upstream of the initial ATG codon was artificially created using PCRs. The stop codon was changed to a BalI site using PCRs. The cDNA was inserted in-frame into pPD95.75 (GFP expression vector; a gift from Andy Fire) using the BamHI and BalI sites (CHB-3::GFP vector). Upstream promoter sequences from *chb-3*, *tax-4*, *odr-1*, *srb-6* and *gpa-9* (2.3 kb for *chb-3*, and approximately 3 kb for the other) were obtained using PCRs and inserted upstream of the *chb-3* coding region of the CHB-3::GFP vector to create *chb-3p*::CHB-3::GFP, *tax-4p*::CHB-3::GFP, *odr-1p*::CHB-3::GFP, *srb-6p*::CHB-3::GFP, and *gpa-9p*::CHB-3::GFP, respectively. The activities of these promoters (other than *chb-3*) have previously been reported [25]-[29] and were confirmed using the GFP expression patterns obtained with each construct. *odr-1p*::CHB-3::mRFP, *srb-6p*::CHB-3::mRFP were created by replacing the GFP coding region with mRFP cDNA.

The GCY-12::GFP construct was made by inserting an 8.1-kb genomic fragment, which contained 3 kb of the promoter region and the entire coding region, in-frame into pPD95.77 using the XbaI and BalI sites. The stop codon was changed to a BalI site using PCRs such that GFP was added to the C terminus of GCY-12. The *odr-1p*::GCY-12::GFP and *srb-6p*::GCY-12::GFP constructs were made by inserting the *odr-1* promoter region (2.4 kb) or the *srb-6* promoter region (3.3 kb) with *gcy-12* cDNA (obtained from the yk316d1 cDNA clone provided by Yuji Kohara; the most 5′ region was supplemented with a product of 5′ RACE) into pPD95.77.

### Generation of transgenic worms

Transgenic strains were generated by standard microinjection methods [60]. Unless otherwise noted, test DNA was injected at 100 ng/µl with a co-injection marker such as *myo-3::gfp* DNA (33 ng/µl, a GFP construct expressed in body wall muscle), *lin-44::gfp* (33 ng/µl, a GFP construct expressed in hypodermal cells at the tip of the tail) or *ttx-3*::mRFP (33 ng/µl, a RFP construct expressed in the AIY neuron). Generally we isolated two to five independent transgenic lines for each injection and confirmed that there were no major differences between them. To compare the expression profiles in different genetic backgrounds, the same extrachromosomal arrays, which were transferred by mating between strains, were used.

For *gcy-12* overexpression analysis, a genomic fragment encompassing 3 kb of the promoter region and the entire coding region of *gcy-12* was sub-cloned into pPD49.26 (expression vector; a gift from Andy Fire), and injected into N2 animals at 150 ng/µl with a injection marker (33 ng/µl *sra-6::gfp*).

### Mapping and cloning of *chb-3*

*chb-3(eg52)* was mapped using the SNP method [61] based on its suppression of the confined tracking phenotype associated with *che-2*. After mating *chb-3(eg52)*;*che-2(e1033)*; *Ex [che-2]* and CB4856 animals, we isolated approximately 100 F2 *chb-3(eg52)*;*che-2(e1033)* animals, which were identified by a cilium defect (indicated by a dye-filling defect) and the wide ranged tracking pattern. Genotype analysis of these lines allowed us to map *chb-3(eg52)* to the left end of LG I. Further mapping was performed based on the Daf-c phenotype. The dauer phenotype was examined in F3 animals instead of F2 offspring of *chb-3(eg52)* and CB4856, because the *chb-3* dauer phenotype was maternally rescued; *chb-3*/+ hermaphrodites did not produce dauer progenies. Thus, F2 progenitors were bleached for their F3 eggs. F3 animals were cultured for 50–60 hours under well-fed conditions and ∼300 dauers were isolated as *chb-3* homozygotes. Genotype analysis of these dauer lines allowed us to map *chb-3* to a ∼820-kb region between the ZC123 marker and the left end of LGI. Six YACs (Y48G1C, Y50C1, Y65B4, Y18H1, Y73A3, and Y2F4) covering this region were introduced separately into *chb-3(eg52)* animals. Two overlapping YACs (Y50C1 and Y2F4) rescued the Daf-c phenotype. Y2F4, a relatively small YAC (190∼230 kb), contained approximately 30 predicted genes. We PCR-amplified genomic fragments covering each gene, and introduced them into *chb-3(eg52)* animals. A 6.5-kb PCR fragment, which was amplified using primers 5′-GATGTTTTCATGGGATGTGCAC-3′ and 5′-TCGGAGATCAATTTTGAGGGC-3′, rescued the Daf-c phenotype of *chb-3(eg52)*. This PCR fragment contained one predicted gene, Y48G1A.3 and ∼1 kb of upstream sequence. A full-length cDNA for Y48G1A.3 was obtained by fusing the yk280C9 clone (provided by Yuji Kohara) and a 5′ fragment (obtained using 5′ RACE). This full-length cDNA also rescued each of the *chb-3* phenotypes examined (see the Results).

To identify the molecular lesion in *chb-3(eg52)*, the genomic coding sequence was amplified from the *eg52* mutant. Three independent PCRs were combined and the products were directly sequenced.

The *chb-3(qj2)* allele was generated using Tc1 transposon insertion and subsequent excision from the *chb-3* coding region basically as described by Zwaal et al. [62]. We used MT3126 *mut-2(r459)*;*dpy-19(n1347)* animals as the mutator line. The isolated deletion allele (*qj2*) was back-crossed 7 times, and maintained as trans-hetero animals with *bli-3* or as the rescued line with an Ex[t*ax-4p*::CHB-3::GFP] transgene, because the Daf-c phenotype in *qj2* homozygotes was severe and few grew into fertile adults. The *qj2* allele lacks 1787 bp beginning in the middle of the 4th exon and ending 61 bp after the 5th exon. This deletion allele appears to produce a CHB-3 protein that lacks the 101 C-terminal amino acids.

### Analysis of *chb-3* transcripts in *chb-3(eg52)* animals using 5′ RACE

Total RNA was isolated from wild-type and *chb-3(eg52)* animals using RNeasy (Qiagen) and reverse transcribed using a *chb-3* gene-specific primer (5′-AGGCGGCATACGTCCTGTTTTC-3′). After TdT tailing, the 5′-end of the *chb-3* cDNA was amplified by using a 5′ RACE system (Invitrogen), an Anchor abridged primer, and a *chb-3* gene specific primer (5′-ATAAGGCGGCAAACATGAGTGG-3′). The PCR products were cloned and sequenced.

## Supporting Information

Figure S1 The GCY-12 levels in the cilia were decreased in *chb-3(eg2)* and *chb-3(qj2)* mutants. *chb-3* mutants were examined in a *che-2(e1033)* mutation background in order to suppress the Daf-c phenotype. Head region of L4 stage animals were shown. Anterior is to the right. The same extrachromosomal array (Ex[GCY-12::GFP]) was transffered by mating from line to line for comparison.
(8.43 MB EPS)

Figure S2 Decreased GCY-12 levels in AWC/AWB cilia were rescued by *chb-3* expression in AWC/AWB neurons. Cilia (nose tips) and cell bodies are marked with circles and squares, respectively (Upper panels) A *chb-3(eg52)* animal carrying the Ex[*odr-1p*::GCY-12::GFP] extrachromosomal array. (Bottom panels) A *chb-3(eg52)* animal carrying two extrachromosomal arrays: Ex[*odr-1p*::GCY-12::GFP] and Ex[*odr-1p*::CHB-3::mRFP]. Ex[*odr-1p*::CHB-3::mRFP] directs the expression of mRFP-tagged, full-length CHB-3 in AWC and AWB neurons. CHB-3::mRFP is distributed throughout the cell, but is excluded from the nucleus. The Ex[*odr-1p*::GCY-12::GFP] array contains *ttx-3p*::mRFP as an injection marker, which was expressed in the AIY neuron (*). Scale bar represents 20 µm.
(4.90 MB EPS)

Figure S3 The expression patterns of *gcy-12* promoter fusion with gfp in wild-type (N2) and *chb-3(eg52)* animals. The same extrachromosomal array (Ex[*gcy-12p*::GFP]) array was used for comparison. The anterior regions of adult animals are shown. No differences in the expression level and pattern between N2 and *chb-3* are seen.
(2.09 MB EPS)

Figure S4 In *chb-3(eg52)* animals, ODR-10 GPCR normally localizes to cilia of AWA sensory neuron (top panel) and AWB sensory neuron (bottom panel). For the localizaiton patterns in wild-type animals, see Dwyer et al., 1998 [45]. GFP patterns also show normal cilium morpholgy of these sensory neurons in *chb-3(eg52)*.
(1.52 MB EPS)

Figure S5 Kymographs depicting GCY-12::GFP particle motility along the dendrite of a phasmid sensory neuron in *chb-3(eg52)* (Upper panels) and *chb-3(eg52)* rescued (Lower panels) animals are shown. The horizontal and vertical axes represent distance and time, respectively. Corresponding lines, with which the velocity and the frequency were analyzed, are also shown. Movies are available upon requests.
(1.57 MB EPS)

Figure S6 The rate of dauer formation at 20°C. The Daf-c phenotype of *chb-3(eg52)* is suppressed by *unc-101(m1)*. Error bars indicate s.e.m.
(0.32 MB EPS)
